# A Robust Protocol for Decellularized Human Lung Bioink Generation Amenable to 2D and 3D Lung Cell Culture

**DOI:** 10.3390/cells10061538

**Published:** 2021-06-18

**Authors:** Mohammadhossein Dabaghi, Neda Saraei, Mabel Barreiro Carpio, Vibudha Nanduri, Julia Ungureanu, Mouhanad Babi, Abiram Chandiramohan, Alexander Noble, Spencer D. Revill, Boyang Zhang, Kjetil Ask, Martin Kolb, Yaron Shargall, Jose Moran-Mirabal, Jeremy Alexander Hirota

**Affiliations:** 1 Department of Medicine, Division of Respirology, Firestone Institute for Respiratory Health, McMaster University, Hamilton, ON L8N 4A6, Canada; dabaghi.sharif@gmail.com (M.D.); n.saraei90@gmail.com (N.S.); chanda15@mcmaster.ca (A.C.); alex_noble@live.com (A.N.); revillsd@mcmaster.ca (S.D.R.); askkj@mcmaster.ca (K.A.); kolbm@mcmaster.ca (M.K.); 2 Department of Chemistry and Chemical Biology, McMaster University, Hamilton, ON L8S 4M1, Canada; barreim@mcmaster.ca (M.B.C.); ungureaj@mcmaster.ca (J.U.); babim2@mcmaster.ca (M.B.); mirabj@mcmaster.ca (J.M.-M.); 3 Department of Chemical Engineering, McMaster University, Hamilton, ON L8S 4L7, Canada; nanduriv@mcmaster.ca (V.N.); zhangb97@mcmaster.ca (B.Z.); 4 School of Biomedical Engineering, McMaster University, Hamilton, ON L8S 4K1, Canada; 5 McMaster Immunology Research Centre, Department of Pathology and Molecular Medicine, McMaster University, Hamilton, ON L8S 4K1, Canada; 6 Department of Surgery, McMaster University, Hamilton, ON L8S 4K1, Canada; shargal@mcmaster.ca; 7 Division of Respiratory Medicine, Department of Medicine, University of British Columbia, Vancouver, BC V6H 3Z6, Canada; 8 Department of Biology, University of Waterloo, Waterloo, ON N2L 3G1, Canada

**Keywords:** decellularization, lung, cell culture, hydrogels, coating, epithelial, fibroblast

## Abstract

Decellularization efforts must balance the preservation of the extracellular matrix (ECM) components while eliminating the nucleic acid and cellular components. Following effective removal of nucleic acid and cell components, decellularized ECM (dECM) can be solubilized in an acidic environment with the assistance of various enzymes to develop biological scaffolds in different forms, such as sheets, tubular constructs, or three-dimensional (3D) hydrogels. Each organ or tissue that undergoes decellularization requires a distinct and optimized protocol to ensure that nucleic acids are removed, and the ECM components are preserved. The objective of this study was to optimize the decellularization process for dECM isolation from human lung tissues for downstream 2D and 3D cell culture systems. Following protocol optimization and dECM isolation, we performed experiments with a wide range of dECM concentrations to form human lung dECM hydrogels that were physically stable and biologically responsive. The dECM based-hydrogels supported the growth and proliferation of primary human lung fibroblast cells in 3D cultures. The dECM is also amenable to the coating of polyester membranes in Transwell™ Inserts to improve the cell adhesion, proliferation, and barrier function of primary human bronchial epithelial cells in 2D. In conclusion, we present a robust protocol for human lung decellularization, generation of dECM substrate material, and creation of hydrogels that support primary lung cell viability in 2D and 3D culture systems

## 1. Introduction

In the last few decades, there has been an effort to develop in vitro lung models to study and understand the mechanism of various lung diseases, such as chronic obstructive pulmonary disease (COPD), idiopathic pulmonary fibrosis (IPF), and cystic fibrosis (CF). For instance, COPD is a progressive lung disease that results in the destruction of the alveoli (emphysema) and inflammation of the airways (bronchitis) [1,2], and it has been estimated to become the leading cause of hospitalization in Canada and the United States by 2022 [3]. The development of new therapeutics for these diseases requires advanced organ models that closely recapitulate the complexity of the human lung. Complementary in vitro, ex vivo, and in vivo animal models have been developed to study and understand various pulmonary diseases [4,5,6,7]. In vitro models (2D cell culture) using epithelial cell lines are high-throughput and robust but are limited by their reliance on cell lines originating from cancer tissue or that have been immortalized. The use of primary human epithelial cells addresses the limitations of these cell sources, yet these human samples are difficult to access, require surgical procedures, and have limited capacity to divide [8]. Unfortunately, even with primary human epithelial cell samples, the current in vitro models frequently do not incorporate extracellular matrix, mechanical forces, and 3D microenvironments that cells experience in normal lung biology [9,10,11]. In vitro organ-on-chip devices have attempted to more accurately model the in situ environment by integrating mechanical forces, including airflow and stretch [12,13,14]. However, most of these models still lack the use of primary cells and tissue-specific ECM components required for cell differentiation, as well as 3D geometries that are important for lung cell differentiation [9,15]. Notably, pulmonary diseases including IPF and COPD frequently cause significant changes and remodeling in the ECM, which is rarely an integrated feature in in vitro models [16,17,18]. Ex vivo models have not been employed extensively and are also limited by access to fresh human lung tissue and face complications to be scaled up to a high-throughput system [19].

In vivo animal models are the most complex systems for preclinical studies, allowing for the whole body and organ-specific physiology outcome measurements, including detailed measures of lung function, pathology, and inflammation in response to various external stimuli and drugs. Unfortunately, in vivo animal models do not perfectly represent human biology and genetics. Although in vivo animal models have been an inseparable part of the drug discovery process, they have constantly failed to provide similar drug responses in preclinical human studies [20]. These limitations have motivated researchers to search for more complex in vitro organ models to fill the gap between the preliminary studies and the preclinical phases.

Tissue-engineered in vitro organ models aim to overcome these limitations by replacing the physiologically inert biomaterials with more biologically active materials that constantly provide biological cues to cells [21,22,23]. Compared to conventional cell culture systems and organ-on-a-chip devices that are considered 2D cell culture systems, these tissue-engineered models are 3D, which can mimic the in situ 3D environment and maintain the phenotype of cells by controlling the polarity of cell-cell and cell-matrix contacts [15,24,25] as well as mechanical properties of their environment [23,26,27] and transport characteristics of important soluble growth factors and cytokines [23]. Among all natural biomaterials for constructing 3D cell culture systems, 3D organ models made of organ-specific decellularized human matrices have attracted attention because they provide the biological cues needed for cells to survive and resemble their native 3D environment in tissues [24,25]. For instance, a recent study showed that cells grown in a mixture of dECM and alginate had higher metabolic activity than the cells grown only in alginate [26]. Various decellularization processes have been developed for different organs hoping to conserve most of the extracellular structure while removing cellular components [24,27,28,29]. The early works focused on developing a procedure to decellularize a whole lung to leave a scaffold behind that could be later seeded by various cells [11,30,31,32]. Recent works have tried to develop techniques to efficiently decellularize lung tissues and solubilize the final product to be used for various applications such as hydrogel formation [31] and coating [33]. However, the tissue source of most studies was from pigs and not from humans, and it has been shown that decellularized human lungs have different characteristics (mechanical properties, ECM components, cells respond to dECM, etc.) compared to decellularized lungs from other species [34]. Balestrini et al. [34] decellularized lungs from rat, pig, primate, and human to investigate DNA content, mechanical properties, and the ECM protein contents. They showed that all decellularized samples had a similar level of collagen, but primate and human decellularized lungs were stiffer and contained a higher level of elastin while GAGs content was lower after the decellularization process. Further, human endothelial cells were cultured onto the decellularized samples to assess the impact of the host on the expression of vascular cell adhesion molecule and activation of nuclear factor-kB, which were significantly lower in human and primate samples.

The absence of a comprehensive standardized decellularization procedure for human lungs prompted our group to explore a variety of reagent compositions and protocols. In this work, we optimized the decellularization process so that the final dECM would be solubilized to form a “bioink” that can be turned into dECM-based hydrogels with mechanical integrity or used for surface modification purposes in Transwell™ insert cell culture systems. In primary experiments, two ionic detergents, sodium dodecyl sulfate (SDS) and sodium deoxycholate (SDC) were used for the decellularization process. We validated the developed decellularization protocol by processing more than 20 different samples. To align with the pursuit of advanced physiologically relevant models, we used primary human lung fibroblast and bronchial epithelial cells instead of cell lines in both 2D and 3D culture systems. We showed that primary human lung fibroblast cells could survive in dECM-based hydrogels and the dECM hydrogels with various concentrations underwent different contraction. Moreover, primary human epithelial cells that were seeded on the surface of dECM hydrogels were viable after a few days of cell culture. Transwell inserts coated with dECM enhanced the cell adhesion and barrier function of epithelial cells. Since this bioink has acceptable gelation property, it can be used for bioprinting application in the future work.

## 2. Materials and Methods

### 2.1. Decellularization

**Scheme 1:**
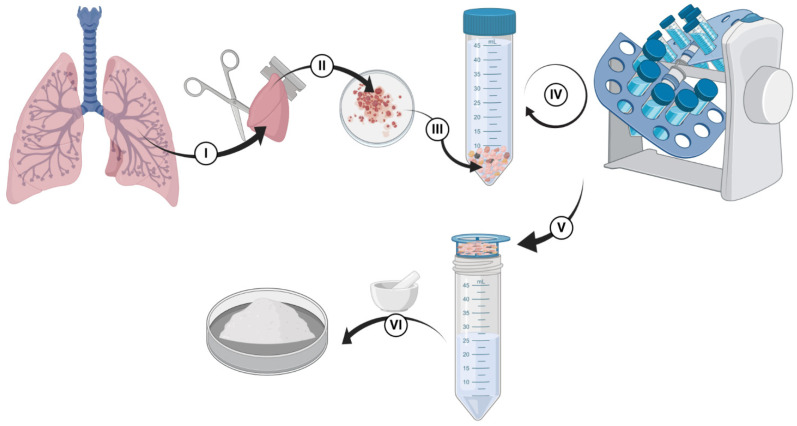
Decellularization process of human lung tissue. (**I**,**II**) tissues were cut into smaller pieces before starting the decellularization process, (**III**) a volume of lung material was transferred into a 50 mL falcon tube for the decellularization process, (**IV**) performing the decellularization steps using one ionic and one non-ionic detergent followed by several washes in PBS, (**V**) removing water from the lung materials before lyophilization, and (**VI**) dry-freezing.

Lung tissue was used from patients who consented to participate in the study, and the protocol was approved by the Hamilton Integrated Research Ethics Board (HiREB-5305-T). All patients were undergoing surgical lung resection as part of their clinical care. During the lung surgical procedure, any tissue remaining following clinical diagnosis and preservation of specimen integrity for a later clinical-pathological assessment and staging was provided to our research team. The decellularization process is shown in Scheme 1 and Figure S1. Small pieces of human lung tissues (Scheme 1I) were received from thoracic resections and transferred in cell culture media (Dulbecco’s Modified Eagle Medium, DMEM). Fresh tissues were moved into a biosafety cabinet for further processing. In the first step, tissues were cut into smaller pieces (1-cm blocks were sufficient for this step) using surgical scissors and razor blades, as shown in Scheme 1II. Representative pieces were stored for histology, protein analysis, and DNA quantifications before starting the decellularization process. Then, tissue blocks were cut into smaller pieces (~2–3 mm slices). A volume of lung material amounting up to 5 mL was transferred into a 50 mL falcon tube (Scheme 1III). If multiple 50 mL falcon tubes were used due to the size of the sample provided—it was ensured that equal amounts of the lung were in each tube as varying the amounts would impact the decellularization steps (e.g., more tissue per fixed amount of reagents would result in less decellularization). For each stage, tubes were filled with a designated reagent of that step (for instance, PBS or Triton X100) up to 40 mL, and it was ensured that the tissue content did not exceed 5 mL. 0.1% Triton X was added to tubes, and they were incubated on a rocker/nutator for 30 min. Tube contents were removed by pouring them through a 100 µm pore cell strainer (Falcon-352,360-Yellow strainer, VWR, Ontario, Canada), the solids recovered into tubes, and the flow through was discarded into a waste container in the biosafety cabinet. Phosphate-buffered saline (PBS) was added to the tubes, and they were agitated on a rocker/nutator for 30 min to wash out the 0.1% Triton X. PBS was removed via straining as above, and 2% SCD was added to each tube. SDC is an ionic detergent that is useful in disrupting cytoplasmic and nuclear contents by detaching protein-protein interactions [30,35]. They were placed on a rocker/nutator and were agitated at 4 °C in a fridge overnight. The day after, the 2% SDC was removed by straining, and PBS with Antibiotic-Antimycotic (Gibco^®^ Antibiotic-Antimycotic 15240062, ThermoFisher, Canada, 100 units/mL of penicillin, 100 µg/mL of streptomycin, and 0.25 µg/mL of Gibco Amphotericin B) was added into tubes, and they were agitated for 24 h (PBS of each tube was exchanged with fresh PBS at least for one more time during this 24-h wash). Then, PBS was removed, and the samples were agitated with autoclaved deionized (DI) water for at least 2 h. Autoclaved DI water was replaced with a 1 M sodium chloride (NaCl) solution, which acts as a hypertonic solution [36,37], and the tubes were agitated on a rocker/nutator for 30 min (Pause point: Washes with water or PBS are very important, and they should not be skipped since they act as hypo-osmotic stress, which helps break cells up to let their content leak out; NaCl will interact with SDC and solidify in the tissue, thereby there need to be extensive washes between the original SDC addition and the NaCl addition). The 1 M NaCl solution was removed via straining, autoclaved DI water was added to the tubes, and they were agitated on a rocker/nutator for 30 min. Water was removed, and 1% Triton X was added to samples, followed by 30 min of agitation on a rocker/nutator. After washing samples with 1% triton X, PBS with antibiotic–antimycotic was added into each tube, and the samples were agitated on a rocker/nutator for 2–3 days. Next, PBS was removed and replaced with autoclaved DI water, and the tubes were rocked for 30 min (Scheme 1IV,V represent all these washing steps with different reagents, PBS, and water). Water was removed from samples using a strainer, and the samples were stored in a −80 °C freezer before being lyophilized. After lyophilization, decellularized tissues were ground using liquid nitrogen and a mortar/pestle to obtain fine dECM powders (Scheme 1VI). The powders were stored in a 4 °C fridge until used for experiments.

### 2.2. Tissue Histology

Before and after decellularization, at least three pieces of intact and decellularized lung tissues were fixed in 10% formalin, followed by ethanol dehydration. The samples were processed for standard hematoxylin and eosin, Masson’s Trichrome, and Elastin van Gieson stains to visualize the removal of cellular components and the perseverance of ECM proteins.

### 2.3. Biochemical Assays

Biochemical assays were performed to determine native tissue and dECM components in samples. All the results were normalized to starting dry tissue weight. Collagen, glycosaminoglycans (GAGs), and elastin present in the samples were quantified using the Sircol, Blyscan, and Fastin kit assays, respectively (Biocolor, UK). DNA was quantified using a Quant-iT Pico Green dsDNA assay kit (Invitrogen, Eugene, OR, USA). All the assays were performed according to the manufacturer’s instructions.

#### 2.3.1. DNA

Dried tissue (2–4 mg) and dECM samples (8–10 mg) were incubated for 3 h at 37 °C in a Proteinase K solution with a concentration of 100 μg/mL (1.5 mL for lung tissue samples and 1 mL for dECM samples). Double-stranded DNA (dsDNA) in the extract was quantified using a Quant-iT Pico Green dsDNA assay kit (Invitrogen, Eugene, OR) according to the manufacturer’s instructions.

#### 2.3.2. Collagen

Dried tissue and dECM samples (3–5 mg) were separately weighted. Tissue samples were washed with PBS at least 2–3 times to remove excess blood and centrifuged. All samples were digested overnight at 4 °C in 1.5 mL of 0.1 mg/mL pepsin solution prepared in 0.5 M acetic acid. Collagen in the extract was quantified with the Sircol collagen assay kit (Biocolor) according to the manufacturer’s instructions.

#### 2.3.3. GAGs

Dried tissue and dECM samples (10 mg) were digested in papain with a final concentration of 125 µg/mL (from papaya latex, it was purchased from Sigma-Aldrich, P4762) extraction reagent (1 mL) in a water bath at 65 °C for 4 h. GAGs content in the extracts was quantified with the Blyscan assay kit (Biocolor) according to the manufacturer’s instructions.

#### 2.3.4. Elastin

Tissue (6–7 mg) and dECM (3–4 mg) samples were digested in 0.25 M oxalic acid (2 mL) at 90–95 °C for 1 h. Three extractions were performed, and the extracts were combined for each sample. Elastin was quantified using the Fastin elastin assay kit (Biocolor) according to the manufacturer’s instructions.

### 2.4. Evaluation of Hydrogel Fiber Orientation Using Scanning Electron Microscopy

**Scheme 2:**
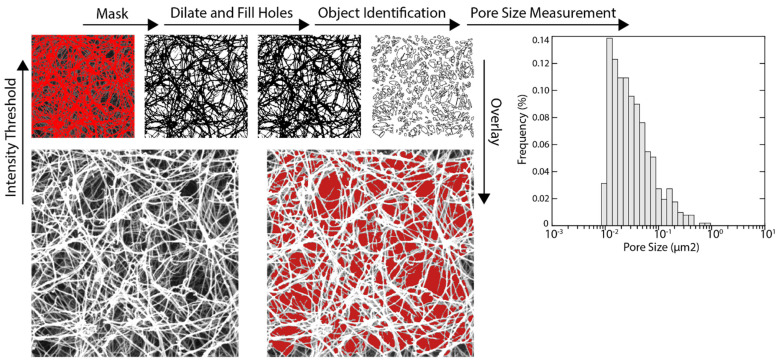
Identification and measurement of pores within dECM SEM images. At least four SEM images were acquired at 25,000× magnification to perform these analyses.

Scanning electron microscopy (SEM) was used to examine the hydrogel fiber network using an established protocol in the literature for dECM hydrogels [38]. dECM hydrogels with concentrations of 12.5, 15, 17.5, and 20 mg/mL were first rinsed with PBS buffer and fixed for 24 h in cold 2.5% glutaraldehyde (Electron Microscopy Sciences, Hatfield, PA, USA) that was prepared in PBS (pH = 7.2). After fixing the dECM gels, they were washed with PBS buffer three times, and the fixed dECM gels were dehydrated in a graded series of ethanol (30, 50, 70, 90, and 100%) for 45 min. After dehydration of the dECM gels, they were stored in 100% ethanol at 4 °C. A critical point dryer was used to gently dry dECM hydrogels. The dried hydrogels were sputter-coated with gold and imaged with a scanning electron microscope (Voltage = 20 kV). The average diameter of fibers for each sample was measured by randomly selecting 20 fibers from 3 SEM images and analyzing them using ImageJ software (National Institutes of Health, Bethesda, MD, USA). SEM images (25,000× magnification) of dECM samples were analyzed using ImageJ to identify and measure pores present within the scaffold. Since dECM pores were represented by regions of low pixel intensity in an SEM image, application of global intensity threshold generated a binary mask that selectively covered porous areas. The mask was dilated, and small holes were filled in order to reduce speckling and improve connectivity between fibrils. Using the Analyze Objects function, individual white spots within the mask that were larger than 0.005 μm^2^ were identified as pores, and their area was measured (Scheme 2).

### 2.5. Human Lung dECM Based Hydrogels

Any noticeable black impurities, potentially carbonaceous remnants from tobacco or occupational exposures from the donors, were manually removed from dECM powders before starting the digestion process. dECM powder with a 22 mg/mL concentration was added to 0.01 N HCl, and pepsin with a 2 mg/mL concentration was dissolved to start digestion of dECM powders. The solution’s pH was checked to ensure that it was somewhere between 2 and 3 (if pH is higher than 3, 1 N HCl was added to lower the pH). The solution was stirred for three days at room temperature (RT). After 24 h, the pH was measured, and it was adjusted if needed. After 72 h, the dissolved dECM was spun down at ~2000–6000 g for 10–15 min. The supernatant was carefully transferred into 15 mL conical tubes, and the remaining non-dissolved materials were discarded. The tubes containing dissolved dECM were placed on ice to be cooled down (a sufficient volume of 10× PBS was placed on ice). Then, 10× PBS (10% of the volume of dissolved dECM) was added to dissolved dECM to reach a final concentration of 20 mg/mL. Next, the pH of dissolved dECM was adjusted to ~7.2–7.4 using 10 N and 1 N NaOH (all steps were performed on ice). To make dissolved dECM with various concentrations, ice-cold PBS (pH = 7.2–7.4) was added to the stock dissolved dECM (concentration = 20 mg/mL) to obtain five different concentrations (20, 17.5, 15, 12.5, and 10 mg/mL). However, the hydrogels with 10 mg/mL concentration were not stable enough in cell culture media and quickly degraded. Therefore, any hydrogels with a lower than 12.5 mg/mL concentration were not included in the cell culture experiments. When air bubbles were trapped in dissolved dECM, dissolved dECM (placed on ice) was degassed in a desiccator for 2 min. To start gelation, dissolved dECM was poured into desired plates or molds and placed in an incubator for ~1 h.

### 2.6. Surface Modification with dECM Precursor

To improve cell attachment and barrier function of primary human bronchial epithelial cells (HBECs) cultured on a porous membrane, dissolved dECM with various concentrations were coated on Transwell™ inserts or polyester track-etched membrane (PETE) with the identical characteristics (pore size of 0.4 µm, 12 microns thick, 2 × 10^6^ pores/cm^2^, 13 mm diameter purchased from Sterlitech, WA, USA). After pepsin digestion of dECM materials, dissolved dECM with a concentration of 22 mg/mL was diluted to lower concentrations (4, 2, and 1 mg/mL) using 0.01 N HCL for surface modification purposes. A sufficient volume of dissolved dECM was added into Transwell inserts or poured onto PETE membranes and stored in a fridge at 4 °C for 24 h. Later, dissolved dECM was aspirated, and the samples were thoroughly rinsed with PBS. Before seeding cells, the samples were sterilized via UV irradiation in a biosafety cabinet.

### 2.7. Turbidimetric Gelation Kinetics

To understand the gelation kinetics of dECM hydrogels, a turbidity assay was performed as previously reported in other work [38,39]. One-hundred microliters (100 µL) of a neutralized liquid dissolved dECM pre-gel solution at various concentrations of 20, 17.5, 15, and 12.5 mg/mL were transferred to 96-well plates (n = 4), and the absorbance (405 nm) was measured every three minutes for 80 min (the plate reader was pre-heated to 37 °C). The measured absorbances were normalized to scale them from 0% (time = 0 min) to 100% (time of maximum absorbances) using the below equation:

$$Ab_{N}=\frac{Ab_{t}-Ab_{0}}{Ab_{max}-Ab_{0}}$$

where $Ab_{N}$ is the normalized absorbance at a given time, $Ab_{t}$ is the absorbance at that time, $Ab_{0}$ and $Ab_{max}$ are the initial and maximum absorbances, respectively. The normalized absorbances were plotted against time, and these curves were analyzed to obtain these parameters: the time to half gelation (t_1/2_), which was defined as the time to reach 50% of the maximum turbidity, the time to get 95% of the maximum turbidity (t_95%_), the gelation rate (S), which was acquired from the maximum slope at t_1/2_, assuming that the turbidity curves were linear before t_1/2_, and the lag time (t_lag_), which was defined as the lag time to start gelation.

### 2.8. dECM Hydrogel Rheology Measurements

Rheological properties of dECM solutions at various concentrations were assessed using a DHR Controlled Stress Single Head CMT rheometer (TA Instruments) with a parallel plate geometry. 500 μL of the dECM solutions were loaded into the gap between the top parallel plate (20 mm in diameter) and the lower Aluminum Peltier Plate (1 mm gap). A gap trim was performed at a 100 μm gap to remove excess material around the edges. The gelation process was studied for the different dECM concentrations by assessing the influence of temperature on the storage, G′, and loss modulus, G″. Temperature ramps were run from 4 to 40 °C, at a ramp rate of 1.2 °C/min, a strain of 0.6%, and an angular frequency of 10 rad/s. The derivatives from each curve were calculated to assess the gelation temperature for each concentration—defined as the point of fastest modulus change. Frequency sweeps were also run after the temperature ramps to precisely determine G′ and G″. The rheometer parameters for the frequency sweep were set to perform an Oscillation Frequency test at 37 °C, with a shear strain of 0.6%, an angular frequency of 0.1–100, and 5 points per decade. The modulus value was averaged from 1–10 rad/s and reported as a single measurement. Each test was performed on three independently prepared samples.

### 2.9. Cell Culture and Staining

Primary human bronchial epithelial cells (HBECs) were isolated from bronchial brushing from consenting donor subjects under approved ethics protocols (HiREB-5099-T) and were expanded into T25 flasks using Pneumacult™ Ex-Plus Basal Media (Stemcell Technologies, Vancouver, BC, Canada) with Pneumacult™ Ex-Plus 50× Supplement, 0.01% hydrocortisone stock solution, and 1% antibiotic–antimycotic. Upon ~90% cell confluency, cells were seeded onto dECM hydrogels or passaged to a T75 flask. We used our well-established cell culture protocols [40] to grow and expand HBECs, and the cells were regularly checked under microscopic, and the TEER measurements were performed as an indicator for the cells’ differentiation.

Primary human lung fibroblast cells (HLFCs) were isolated from lung tissues and expanded in DMEM containing 10% fetal bovine serum (FBS) and 1% penicillin-streptomycin. Polydimethylsiloxane (PDMS) was used as a mold to form dECM hydrogel disks. PDMS monomer and curing agent were mixed at a ratio of 10:1, degassed in a desiccator for ~30 min, and cured at 65 °C overnight. Once PDMS was fully cured, it was punched using a biopsy punch with a diameter of 12 mm. PDMS molds were rinsed with 70% ethanol, dried in a biosafety cabinet, and autoclaved before pouring dECM hydrogels. Concentrated HLFCs were suspended in precursor dECM hydrogel solutions (20, 17.5, 15, and 12.5 mg/mL) so that the final cell density of 4 × 10^6^ cells/mL was achieved. Cells were gently and thoroughly mixed in precursor dECM hydrogel solutions and poured into PDMS disks. After one hour of incubation at 37 °C to form dECM hydrogels, DMEM media was added to each disk containing dECM hydrogels and incubated overnight. The day after, the dECM hydrogels with HLFCs were removed from the PDMS disks and transferred to 6-well plates, and immersed in DMEM.

Calcein AM dye (5 μM, Invitrogen, Carlsbad, CA, USA; purchased from Thermo Fisher; product number: C3100MP, Ottawa, ON, Canada) was used to stain the cells. Samples (dECM coated membrane)

### 2.10. Contraction of dECM Hydrogels

Primary human fibroblast cells were seeded within the bulk of dECM hydrogels with four concentrations of 12.5, 15, 17.5, and 20 mg/mL, and the change in diameter was quantified using macroscopic image analysis over five days (n = 5). dECM hydrogels were freely floating inside their wells (6-well plates were used), and DMEM cell culture media and the samples were imaged daily. Unseeded dECM hydrogels for each concentration were prepared and examined as controls. The collagen solution used in this study was PureCol^®^ Type I Collagen Solution from bovine with a concentration of 3 mg/mL and was purchased from Advanced Biomatrix.

### 2.11. Transepithelial Electrical Resistance (TEER) Measurements

Transwell inserts were coated with dECM solutions using three concentrations and seeded with HBECs at a cell density of 200,000 cells/cm^2^. Once the cells reached ~100% confluency, the apical medium was removed, and the cells were basally fed to establish an air-liquid interface (ALI, this was called day 0 of ALI). After 24 h, the cells were basally fed with 750 µL PneumaCult-ALI Basal medium (StemCell Technologies; catalog number 05001, Vancouver, British Columbia, Canada) with PneumaCult-ALI 10× supplement, PneumaCult-ALI Maintenance 100× Supplement, 1% antibiotic-antimycotic, 0.5% hydrocortisone stock solution, and 0.2% heparin solution (StemCell Technologies; catalog number 7980) to support development and differentiation of a pseudo-stratified epithelial culture (day 1 of ALI). Transwell cell cultures were fed from the basal compartment every day, and TEER was measured after adding 200 µL PBS to the apical compartment [40]. TEER measurements were conducted every day until a peak was reached, and the measurements started to drop.

### 2.12. Dextran Permeability Assay

HBECs seeded on Transwell inserts coated with dECM were maintained at ALI upon reaching their maximum TEER values. Then, their permeability to 4 kDa FITC-dextran in PBS (Sigma-Aldrich, Mississauga, ON, Canada) was assessed. A total of 2 mg/mL of 4 kDa FITC-dextran in PBS was added to the apical side of each Transwell insert while 600 µL of the cell culture media was added to the basal compartment. Transwell inserts were incubated for 24 h [33]. The next day, 100 µL was taken from the basal culture media, and fluorescent intensity was measured by a microplate reader (the excitation wavelength was 490 nm and the emission wavelength was 520 nm).

### 2.13. Statistical Analysis

GraphPad Prism 9 was used to perform all statistical analyses (GraphPad Headquarters, San Diego, CA, USA). An unpaired *t*-test was conducted to investigate the significance of the differences in DNA, elastin, collagen, and GAGs contents before and after the decellularization process. A two-way ANOVA was performed to investigate differences in barrier function of HBECs seeded on non-coated and coated Transwell Inserts.

## 3. Results

### 3.1. Histologic and Biochemical Matrix Analysis

**Figure 1:**
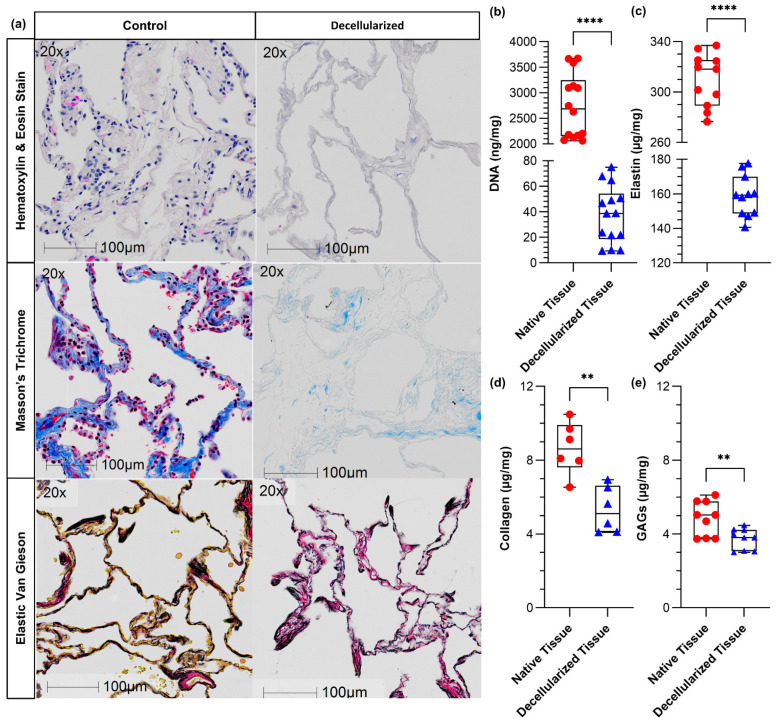
Histologic and biochemical matrix analysis for decellularized human lung tissues: (**a**) hematoxylin and eosin (H&E) images confirmed the removal of the cellular components, (**b**) the quantified double-stranded DNA content using PicoGreen assay before and after decellularization showing that the process was effective, (**c**) the soluble elastin content using The Fastin™ Elastin Assay before and after decellularization, (**d**) the soluble collagen content using The Sircol™ Collagen Assay before and after decellularization, and (**e**) the quantification of soluble GAGs concentration using The Blyscan™ Glycosaminoglycan Assay before and after decellularization. ** *p* ≤ 0.01 and **** *p* ≤ 0.0001. n = 14 from 5 individuals, n = 11 from 5 individuals, n = 6 from 3 individuals, and n = 9 from 3 individuals.

Histological analysis was performed to visualize the removal of cellular components, especially nucleic acid material, and investigate the disruptive impact of the detergents used in the decellularization process on ECM proteins (Figure 1a). All images confirmed a noticeable loss of the double-stranded DNA. The dsDNA concentration was also quantified using the PicoGreen assay, indicating that dsDNA was decreased from 2700 ± 600 (ng of dsDNA/mg of dried tissue) to 40 ± 20 (ng of dsDNA/mg of dECM), which is an acceptable reduction suggested in the literature [15,41] (Figure 1b). Masson’s Trichrome and Elastin van Gieson stains qualitatively showed collagen and elastin were largely retained (more than 50%), albeit with some visible loss after the decellularization process. Quantitative analysis of collagen and elastin confirmed the reduction in both soluble elastin and collagen (Figure 1c,d). The concentration of soluble GAGs was measured using a colorimetric assay, showing a significant reduction in the GAGs content after the decellularization process (Figure 1e), consistent with the trend observed for other ECM proteins.

### 3.2. Turbidimetric Gelation Kinetics

**Figure 2:**
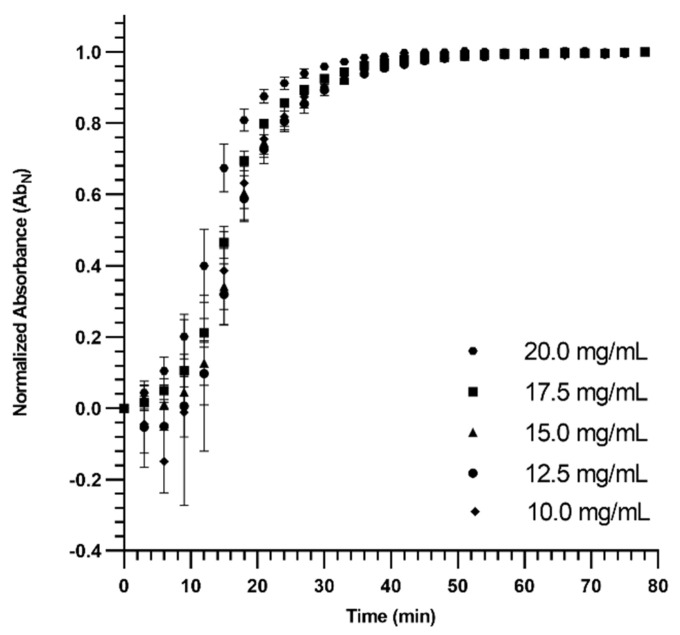
Turbidimetric gelation kinetics of dECM hydrogels with various concentrations. Until the curves plateaued (Time ~57 min), a significant difference between dECM hydrogels with the concentration of 20 mg/mL with at least one of the other concentrations was observed.

The turbidimetric gelation kinetics of dECM hydrogels were spectrophotometrically studied for various concentrations (Figure 2). The related gelation parameters were calculated as presented in Table 1 and Figure S2 (slope, time to 50% gelation, time to reach 95% maximum turbidity, and the lag time). The gelation started with a lag time for all concentrations, and the lag time for higher concentrations (20 and 17.5 mg/mL) was lower compared to the other concentrations. Except for dECM hydrogels with 20 and 17.5 mg/mL concentrations, the slope (the rate of gelation) was not significantly affected by concentration. The time to reach 50% of gelation, the time to 95% of the maximum turbidity, and the lag time followed a similar trend as the slope of the gelation. These results indicated that the gelation potential increased by increasing the concentration of dECM pregel solution when the concentration was greater than 15 mg/mL.

### 3.3. dECM Hydrogel Rheology

**Figure 3:**
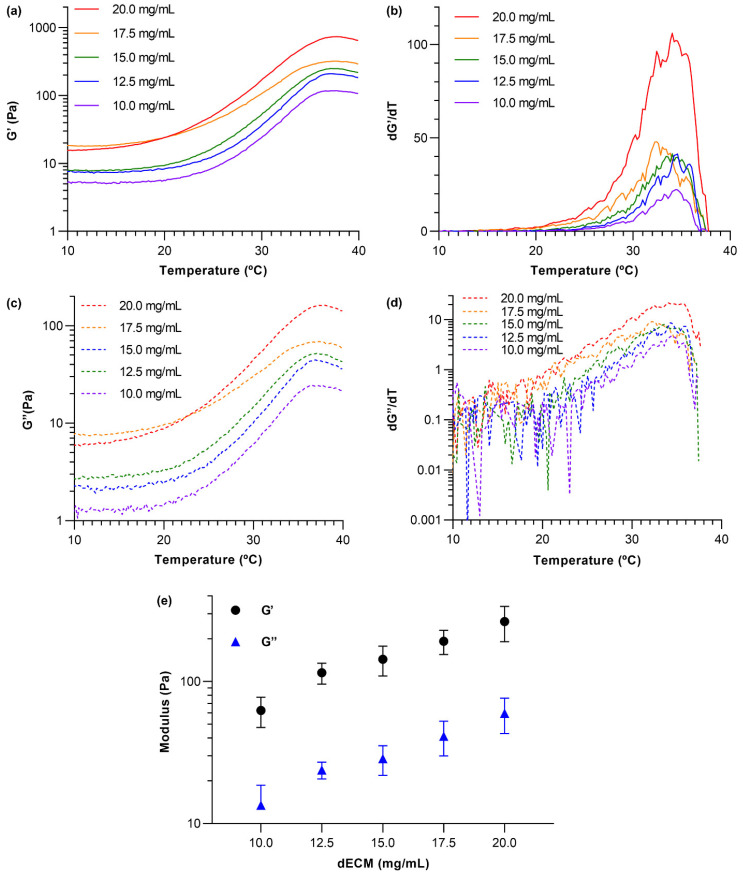
Rheological measurements of dECM hydrogels at different concentrations. (**a**) The storage modulus (G′) was measured as a function of temperature for dECM at various concentrations, (**b**) derivative plot of data presented in (**a**), (**c**) the loss modulus (G″) was measured as a function of temperature for dECM at various concentrations, (**d**) derivative plot of data presented in (**c**), (**e**) the average G′ and G″ of dECM hydrogels at different concentrations (statistical analysis and *p*-values can be found in Figure S3 in the supplementary). N = 3 independently prepared hydrogels and error bars show standard deviation.

A parallel plate rheometer was employed to measure the viscoelastic properties of dECM hydrogels at five different concentrations. The storage (G′) and loss modulus (G″) of dECM hydrogels were measured as the temperature was ramped from 4 to 40 °C, and the derivatives of the modulus plots were calculated as depicted in Figure 3 (a summary of statistical analysis is presented in Figure S3). As the temperature increased, both storage and loss modulus increased and plateaued around ~37 °C, confirming that neutralized dissolved dECM solutions turned into gels. In addition, the ratio of the storage modulus to the loss modulus increased as the temperature was raised, reaching a value of ~5 at full gelation. This suggests that as the dECM gel solutions gelled, they increasingly exhibited behavior characteristics of elastic solids (Figure 3a,c). Derivatives of the modulus versus temperature plots (Figure 3b,d) were used to determine the approximate gelation temperatures of the dECM hydrogels, which are represented by the peaks in the dG′/dT and dG″/dT graphs. These results indicated that the gelation temperature for all dECM hydrogels was around 34 °C. Finally, frequency sweeps at a constant temperature of 37 °C, constant shear of 0.6%, and angular frequencies ranging from 0.1–100 rad/s were acquired to evaluate the storage and loss moduli of the gelled materials. The moduli recorded for frequencies from 1–10 rad/s from sweeps obtained from three independently prepared samples for each dECM concentration were used to calculate average G′ and G″ values (Figure 3e). Both storage and loss moduli increased as dECM concentration increased. This behavior can be attributed to a higher density in the crosslinked network within the dECM hydrogels due to higher protein concentrations.

### 3.4. Macroscopic and Microscopic Appearance of dECM Hydrogels

**Figure 4:**
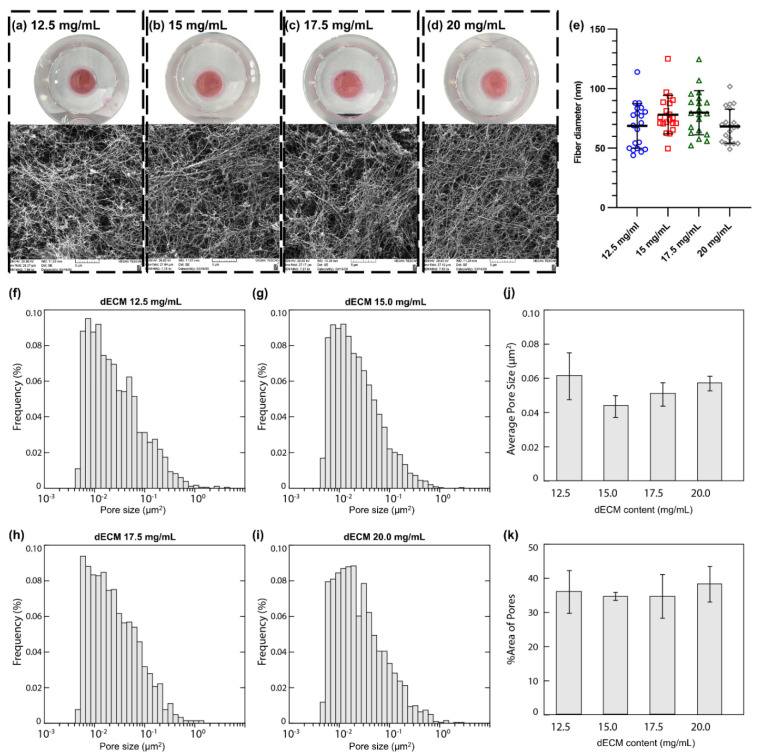
Macroscopic images of dECM hydrogels and corresponding SEM images for concentrations of: (**a**) 12.5, (**b**) 15.0, (**c**) 17.5, and (**d**) 20 mg/mL. (**e**) Fiber diameter analysis of dECM hydrogels. Pore size distribution for dECM hydrogels with the concentration of (**f**) 12.5, (**g**) 15.0, (**h**) 17.5, and (**i**) 20.0 mg/mL. (**j**) The average pore size for dECM hydrogels with various concentrations. (**k**) Area % of pores for dECM hydrogels with various concentrations. The pore size distribution of each dECM sample was based on at least four SEM images acquired at 25,000× magnification.

In this work, dECM hydrogels with various protein concentrations were prepared, and their stability and capabilities to be manipulated in cell culture media were examined. A wide range of concentrations for hydrogels made of decellularized lung tissues (human or animals) has been reported in the literature [39,40,42]. Therefore, we aimed to examine dECM hydrogels with concentrations from 2 to 20 mg/mL to determine an optimum range for human lung dECM hydrogels. Initially, dECM hydrogels with lower concentrations (ranging from 2 to 8 mg/mL) were prepared and manipulated in cell culture media with spatulas to investigate their stability (data not presented here). Since these hydrogels were not mechanically robust to maintain their physical appearance in cell culture media and disintegrated into small pieces or fully/partially dissociate in the media, they were not further studied in the present work. Hydrogels with concentrations greater than 10 mg/mL were more physically robust and easier to handle. However, some of the dECM hydrogels with a 10 mg/mL concentration lost their integrity at the edges of the gels, or small pieces came off from the edges after being manipulated with a spatula. As a result, the lowest concentration of dECM hydrogels with sufficient mechanical and physical stability was determined to be 12.5 mg/mL. Figure 4a–d shows macroscopic images of the dECM hydrogels at four concentrations. The hydrogels were fixed and dehydrated to assess the fiber network formation. SEM images showed the formation of random fiber networks for dECM hydrogels with no preferred orientation or organization at all four concentrations (Figure 4a–d). The diameter of fibers varied from ~40 to ~120 nm, and no significant difference among the concentrations was observed (Figure 4e). Pore size distribution for each dECM hydrogel was evaluated using the custom image analysis algorithm as depicted in Figure 4f–i. Then, the average pore size (Figure 4j) and the percentage of the pore areas (Figure 4k) were calculated, showing no significant difference among all four concentrations. Overall, the average pore size was ranged from ~0.04 to 0.08 µm^2,^ and the pores covered 30% to 40% of the total area.

### 3.5. In Vitro Cell Culture and Viability Assessment

**Figure 5:**
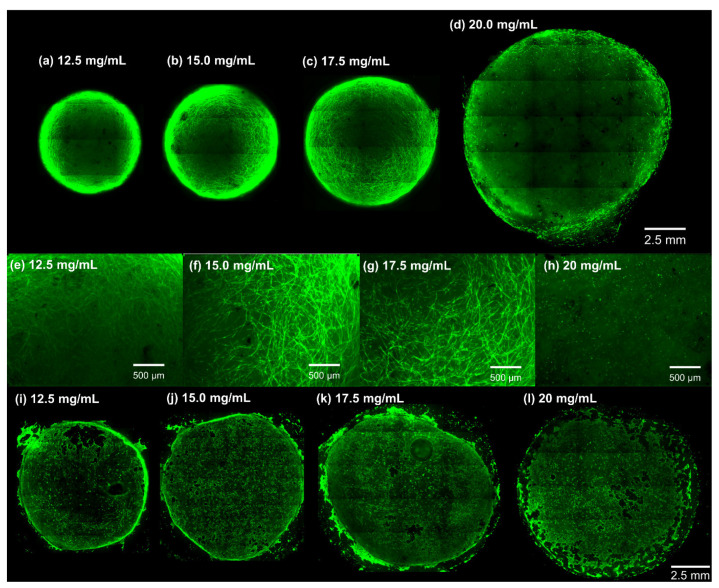
Viability assays of cells incubated inside and atop dECM hydrogels using Calcein AM dye. (**a**–**d**) The viability imaging of primary human fibroblast cells (HFLCs) after 5 days of culture within dECM. Hydrogels with concentrations < 20 mg/mL underwent contraction, and their final diameter decreased significantly compared to their initial size. (**e**–**h**) Close up microscopy images of HLFCs cultured within dECM hydrogels. (**i**–**l**) The viability imaging of primary human bronchial epithelial cells after 4 days of culture on top of the dECM hydrogels’ surface.

A viability assay was performed using Calcein AM staining for both primary HLFCs cultured within dECM hydrogels, and HBECs seeded on the surface of the hydrogels (Figure 5 and Figure S4). Calcein AM staining qualitatively demonstrated that HLFCs cultured within dECM hydrogels made at all concentrations were viable after 5 days of culture. Although all dECM hydrogels were cultured with the same cell density (4 × 10^6^ cells/mL), a change in the cells’ morphology was observed along with a marked dECM hydrogel contraction. Cells had a spindle-shaped morphology in the gels at concentrations < 20 mg/mL (Figure 5a,b). On the other hand, the cells in the dECM hydrogels with a concentration of 20 mg/mL had a spherical morphology (Figure 5a–h). These results confirmed that dECM hydrogels with a 20 mg/mL concentration presented a different, more dense and rigid microenvironment that impacted cell biology in the dECM hydrogels. Additionally, HBECs were seeded on the surface of dECM hydrogels with various concentrations. The hydrogels were stained with Calcein AM and imaged. The Calcein AM staining indicated that the HBECS covered the surface of the dECM hydrogels and were viable after 4 days of culture (Figure 5i–l).

### 3.6. Contraction of dECM Hydrogels

**Figure 6:**
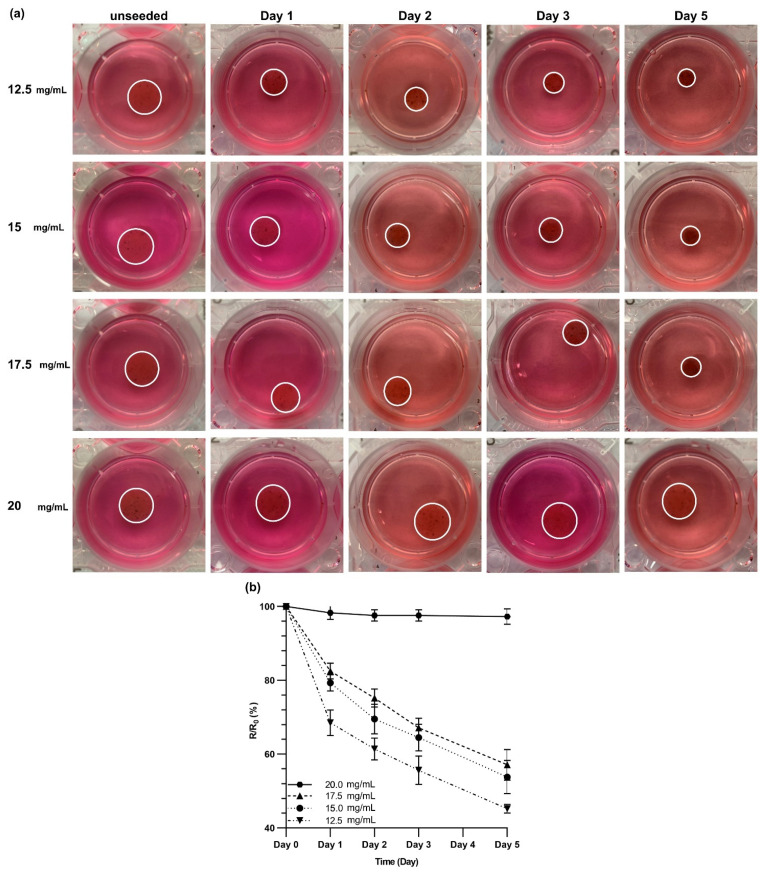
Contraction of dECM hydrogels over five days of 3D culture. (**a**) Primary human fibroblast cells were cultured within dECM hydrogels with various concentrations, and these were imaged over five days to measure the decrease in the diameter. (**b**) The ratio of the measured radius to the radius of the unseeded control gels at the given day. This graph shows a significant reduction in the diameter of dECM hydrogels with concentrations of 12.5, 15, and 17.5 mg/mL. Sstatistical analysis and *p*-values can be found in Figure S5 in the supplementary.

HLFCs were cultured within dECM hydrogels with various concentrations of 12.5, 15, 17.5, and 20 mg/mL, and the contraction of the gels was monitored over 5 days of culture (Figure 6a and the statistical analysis is shown in Figure S5). The dECM hydrogel contracted more as the concentration of dECM was decreased. The dECM hydrogels with a 20 mg/mL concentration did not show any noticeable contraction over 5 days of culture. The hydrogel contraction was quantified by calculating the ratio of the radius of the gel to the radius of the unseeded gel (Figure 6b). The rate of the contraction was the greatest in the first 24 h of culture for dECM hydrogels with concentrations of 12.5, 15, and 17.5 mg/mL. After 24 h, the contraction rate almost became constant for these three concentrations. The dECM hydrogels with concentrations of 12.5 and 15 mg/mL underwent the same contraction with the final ratio (R/R_0_) of 54 ± 5% and 57 ± 4%, respectively (Figure 6b). However, the R/R_0_ ratio was 45 ± 1% for the dECM hydrogels with 12.5 mg/mL concentration. It can be concluded that altering the concentration of dECM hydrogels would impact the physical and biological properties of dECM hydrogels and can be used to control the microenvironment of cells.

### 3.7. The Impact of dECM Coating on Cell Adhesion and Proliferation

**Figure 7:**
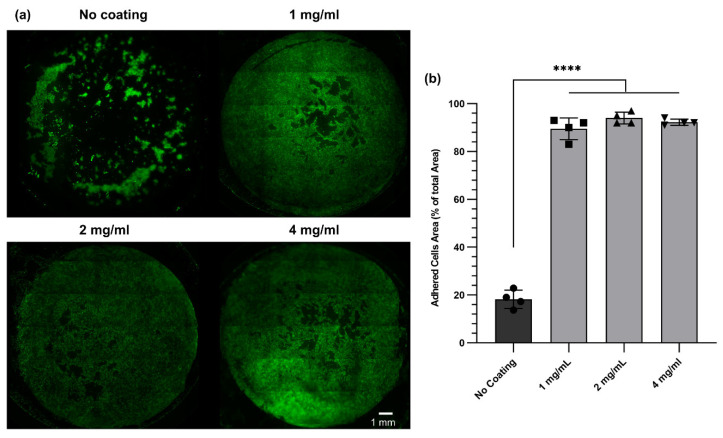
Viability assays of human bronchial epithelial cells cultured for 7 days on dECM-coated polyethersulfone (PES) membranes. (**a**) Viability images of the cells for non-coated and coated membranes. (**b**) The adhered cells’ area as a percentage of the total surface area of the membrane (diameter of PES membranes was 13 mm) was evaluated using fluorescence microscopy images and analyzed in ImageJ software. **** *p* ≤ 0.0001.

Coating a cell culture surface with an ECM-based protein could enhance cell adhesion and proliferation, as has been reported in previous studies [33,41,43,44]. Among all ECM proteins, collagen is the most commonly used protein in cell culture. Nonetheless, recent studies have shown that a dECM-based coating could enhance cell adhesion and functionality to a greater extent than a simple collagen coating [33]. Therefore, we used digested dECM solutions to coat Transwell™ inserts and polyester membranes. To investigate the effect of dECM coating on primary HBEC adhesion and proliferation, polyester membranes with identical properties as Transwell™ inserts were coated with dECM solution at three concentrations (4, 2, and 1 mg/mL). After 7 days of culture, the cells were stained with Calcein AM (Figure 7a). The staining revealed that the cell attachment and proliferation were improved for all coated membranes compared to non-coated membranes. This was in agreement with our expectations and the literature. The adhered cell area was also quantified using ImageJ software, showing a significant difference between dECM-coated samples and non-coated samples (Figure 7b).

### 3.8. The Barrier Function of Primary Human Bronchial Epithelial Cells

**Figure 8:**
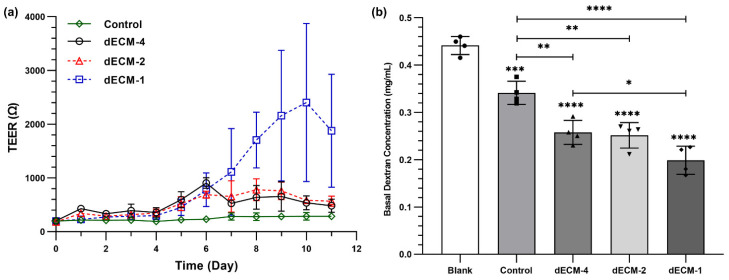
Barrier function of human primary epithelial cells grown at ALI. (**a**) TEER measurement for coated and non-coated Transwell membrane. (**b**) Permeability testing of the cells using fluorescent dextran (4 kDa). * *p* ≤ 0.05, ** *p* ≤ 0.01, *** *p* ≤ 0.001, and **** *p* ≤ 0.0001. Control represents cells seeded on Transwell inserts without coating.

The barrier function of primary HBECs was evaluated by measuring TEER values and a permeability assay using FITC-dextran (4 KDa). Typically, HBECs with a passage number of two cultured at ALI would not reach high TEER values (all TEER measurements were below 400 Ω). However, dECM coating improved the TEER measurements over 11 days of ALI culture (Figure 8a,b). In the first week of ALI culture, TEER values gradually increased for all concentrations tested (1, 2, and 4 mg/mL). Interestingly, the concentration coating did not impact TEER in the first seven days of ALI culture, but the concentration played a role in the second week of ALI culture. TEER values for dECM coated Transwell™ insert with two concentrations of 4 and 2 mg/mL reached a peak and plateaued around 7 days of ALI culture. However, TEER measurements for the coated samples with a 1 mg/mL concentration still increased and peaked at day 10. These results revealed that dECM coating enhanced HBECs barrier resistance, and a lower concentration (1 mg/mL) could be optimal for cell differentiation and barrier function formation.

FITC-dextran with a concentration of 2 mg/mL was added to the apical side of HBECs cultured under ALI, and samples were collected from the basal compartments after 24 h to quantify the concentration of dextran (Figure 8). When there were no cells added on the Transwell™ inserts, the dextran concentration in the basal side was 0.44 ± 0.02 mg/mL. After culturing HBECs under ALI conditions, dextran concentration in the basal side significantly dropped to 0.34 ± 0.02 mg/mL. Dextran concentration in the basal side for the dECM coated samples with coatings form solutions concentrations of 4, 2, and 1 mg/mL was 0.26 ± 0.02, 0.25 ± 0.02, and 0.2 ± 0.03 mg/mL, respectively (Figure 8b). The permeability assay with dextran also confirmed that a concentration of 1 mg/mL improved the barrier function of HBECs compared to two other concentrations.

## 4. Discussion

In this study, we provide a validated workflow for human lung decellularization, dECM isolation, and dECM solubilization for applications in advanced 2D and 3D culture of lung cells, including primary fibroblasts and bronchial epithelial cells. Enzymatic solubilization of dECM with pepsin to create dissolved dECM enabled both 2D coatings and 3D structures under controlled temperature conditions for advanced cell cultures.

The conventional cell culture systems mainly focus on studying the genetics and biochemistry aspects of cell biology without considering how the mechanical properties of the cell culture matrix impact cell behavior. As a result, some earlier studies used polyacrylamide hydrogels coated with ECM-based components that were engineered to have various mechanical properties to understand the impact of the substrate stiffness on cell attachment, migration, and spreading [45]. Nevertheless, the nature of these synthetic hydrogels is significantly different from native ECM, which can cause undesired changes in cell fate [46]. Moreover, the majority of these hydrogels are biologically inert and do not provide natural binding sites for cell attachment and movement [46]. To address this shortcoming, ECM-based materials have been used as a coating in 2D cell culture platforms or tissue-engineered hydrogels. The decellularization process has been recognized as a promising tool to preserve vital ECM components that can be utilized in developing tissue-engineered surfaces or hydrogels [47]. This was the driving force behind our work, aiming at establishing a robust and reproducible decellularization tissue process for human lung tissues.

Hydrogels derived from decellularized tissues can mimic the biochemical composition of the native tissues at a higher degree of complexity compared to those hydrogels that are only made of one ECM protein. Since the earliest attempts to decellularize tissue, preserving the critical components of ECM (i.e., collagen, elastin, GAGs, growth factors, and proteoglycans) was the key to develop a successful decellularization process because these ECM components define the biochemical properties, nanostructure, and biological complexity of the native tissue of the interest [24]. Therefore, any decellularization process should be harsh enough to remove the cellular components with minimum leftover, but it should be delicate enough to retain ECM components that are still bioactive. Histological analysis before and after the decellularization process confirmed that the developed protocol was successful to fully decellularize all samples processed, which was further supported by the biochemical quantification of DNA, elastin, collagen, and GAGs contents. While our protocol results in a reduction in the soluble elastin, collagen, and GAGs content, the result of multiple series of detergent washes, each ECM component was still detectable after complete removal of nucleic acid material. This is consistent with other decellularization protocols reported for various organs [37,38].

A turbidimetric kinetic study was performed to assess the gelation behavior of dECM solutions. Solutions with concentrations ranging from 2 to 20 mg/mL were studied and compared with the gelation behavior of soluble collagen at a concentration of ~2.5 mg/mL. Our early investigations showed that neutralized dECM pregel solutions with concentrations below 10 mg/mL would not form stable gels. Figure 2 demonstrates the turbidimetric kinetic analysis of the dECM solutions at five different concentrations. Except for the highest concentrations (17.5 and 20 mg/mL), other dECM solutions showed a similar gelation behavior. The dECM solutions with a concentration of 20 mg/mL demonstrated faster gelation compared to other concentrations, a feature that might be leveraged for applications, such as bioprinting, that require fast gelation. Another study performed by Pouliot et al. [39] investigated the gelation kinetics of porcine lung dECM solutions based on pepsin digestion time. They concluded that longer digestion time led to slower gelation. Since the main focus of this analysis was to understand the gelation kinetics of dECM hydrogels based on concentration rather than digestion time, all samples were digested for 72 h. Therefore, a shorter digestion time and a higher concentration of dECM solution can be used to achieve faster gelation when needed.

The biocompatibility of dECM hydrogels was assessed with two primary lung cell types. First, we examined how dECM hydrogels could support the survival of HLFCs after multiple days in 3D culture. Cells in all four tested dECM hydrogels were viable and responded differently to the dECM hydrogel concentration as a biomechanical cue. It is known that fibroblasts have a spindle-shaped morphology with noticeable contractile capabilities. Varying mechanical or biochemical cues may impact fibroblast morphology [42,48]. Here we studied the impact of a biomechanical cue (density of the dECM gel network) on primary human lung fibroblasts by varying dECM concentrations. We noticed that the dECM hydrogels within which HLFCs were cultured underwent rapid contraction one day after hydrogel formation, with the exception of dECM hydrogels with a concentration of 20 mg/mL (Figure 5 and Figure 6), which did not show significant contraction when compared to the unseeded control. However, the rate of contraction differed based on concentration and days of culture. dECM hydrogels with a concentration of 12.5 mg/mL showed the highest contraction, as depicted in Figure 5b. These results confirmed that fibroblasts are influenced by their microenvironment, and the concentration of ECM may impact their ability to remodel their local environment. This aspect of dECM hydrogels could be leveraged to model mechanisms of chronic lung diseases such as pulmonary fibrosis, where ECM deposition and fibroblast biology are dysregulated [49,50,51].

Any change at the cell culture interface may affect cell morphology or phenotype. Surface patterning with different geometries from the nanoscale to microscale is able to influence cellular behavior [52]. Moreover, modifying the cell culture surface with ECM proteins can have an effect on cell behavior, their survival, and proliferation or fate. This response can be regulated by varying the composition of ECM proteins to mimic a more physiological microenvironment for cells. For the epithelial cell culture system, coating the surface with ECM proteins can enhance cell attachment and barrier function [32,33,53]. Therefore, we coated Transwell inserts with dECM, which was processed using our decellularization and solubilization protocol, to improve the cell adhesion (in submerged cell culture model and ALI culture) and barrier function of HBECs under ALI conditions. Polyester membranes with identical physical properties as Transwell membranes were coated with dissolved dECM and cultured with HBECs. After 7 days of culture, noticeable cell attachment and proliferation were observed compared to the uncoated polyester membranes. TEER measurements (Figure 8a) and FITC-dextran permeability assay (Figure 8b) demonstrated that coating cell culture surfaces with dECM materials plays an important role in enhancing primary epithelial cell barrier function. Although cell staining with Calcein AM did not show any appreciable difference in cell proliferation, the barrier function of the cells was affected by the concentration. Overall, these results suggest that dECM can be used to coat the cell culture substrates and improve cell adhesion and functionality. As shown in this study, rendering the cell culture surfaces with ECM-based proteins had a large impact on cells’ responses. Physiologically relevant cell culture platforms have been developed and showed that surface curvature influences alveolar cell behavior [9,54]. In these studies, rigid synthetic polymers have been used to mimic the desired curvature. An interesting approach would be modifying the surface of such curved structures with dECM to have a complex and physiologically relevant cell culture system.

Bioinks derived from solubilized dECM have emerged as a new category of biomaterials for bioprinting. The main challenge of using solubilized dECM for bioprinting is its slow gelation and poor mechanical stability. To be able to print a 3D structure, the printing material should be solidified quickly after deposition on the printed surface to retain the desired shape. Since solubilized dECM alone is not a suitable material for bioprinting, a new strategy has been developed to capture the various benefits of dECM in a bioink while addressing the shortcoming of their printing. A hybrid bioink [26] or a mixture of solubilized dECM with another printable biomaterial [55] has been developed to successfully print more complex constructs that contain biochemical and biological cues thanks to having dECM. Although our dECM hydrogels demonstrated good mechanical stability over a few days of culture, they also exhibit slow gelation kinetics, as reported by others [26,55]. Therefore, a similar strategy can be used to reinforce dECM for bioprinting. Different printable biomaterials such as gelatin methacryloyl (GelMA) [56,57], collagen [58], Pluronic [59], or a mixture of these candidates [60] can also be investigated in the future as a reinforcement bioink for bioprinting dECM-based 3D structures.

## 5. Summary

In this work, human lung tissues have been decellularized and enzymatically solubilized to form a bioink that can be used for 2D and 3D cell culture applications. Various concentrations of dECM solutions were investigated to form dECM-based hydrogels with suitable mechanical stability. The cell viability of these hydrogels was studied using primary human lung fibroblast cells and primary human bronchial epithelial cells, showing that the cells could survive and proliferate on the surface of the hydrogels or within the gels. Moreover, the results indicate the dECM solution could be used to coat Transwelll inserts to enhance cell adhesion and growth. Such a dECM-based bioink could potentially be utilized to fabricate in vitro lung constructs for modeling and studying various pulmonary diseases in the future.

## Figures and Tables

**Table 1:** Summary of gelation kinetics parameters for dECM hydrogels. Statistical analysis and *p*-values can be found in Figure S2 in the supplementary.

Parameter	Slope		t_1/2_		t_95%_		t_lag_	
Concentration	Average	±SD	Average	±SD	Average	±SD	Average	±SD
10.0 mg/mL	0.034	0.007	20.79	5.21	36.26	1.07	9.57	2.78
12.5 mg/mL	0.034	0.002	19.83	1.42	38.22	2.01	9.98	3.56
15.0 mg/mL	0.030	0.005	20.39	3.03	36.89	2.43	9.15	1.69
17.5 mg/mL	0.035	0.003	17.14	0.62	33.53	2.20	6.10	2.24
20.0 mg/mL	0.041	0.004	14.16	1.54	29.23	1.17	3.52	1.19

## Data Availability

The data that support the findings of this study are available from the corresponding author upon reasonable request.

## Supplementary Materials

The following are available online at https://www.mdpi.com/article/10.3390/cells10061538/s1, Figure S1: Macroscopic images from various decellularization process steps, Figure S2: A summary of statistical analysis and p-values for calculated turbidimetric gelation kinetics parameters in Table 1, Figure S3: A summary of statistical analysis and p-values for G’ and G’’ in Figure 3e, Figure S4: Viability assays of cells incubated inside dECM hydrogels using the live and dead assay, Figure S5: (a) Heat map of the p-values for each hydrogel over five days: this graph shows if the change in the diameter of the hydrogel is significantly changed over time and (b) heat map of the p-values analysis for hydrogels compared to each other over five days of 3D culture.
