# Supplementary material for: A Robust Protocol for Decellularized Human Lung Bioink Generation Amenable to 2D and 3D Lung Cell Culture
Source: Cells. 2021 Jun 18;10(6):1538. doi: 10.3390/cells10061538 (PMC8234522; doi:10.3390/cells10061538)
Supplement: Supplementary file 1 [file cells-10-01538-s001.zip › cells-1262441-supplementary.pdf]

A robust protocol for decellularized human lung bioink generation amenable to 2D and 3D lung cell culture

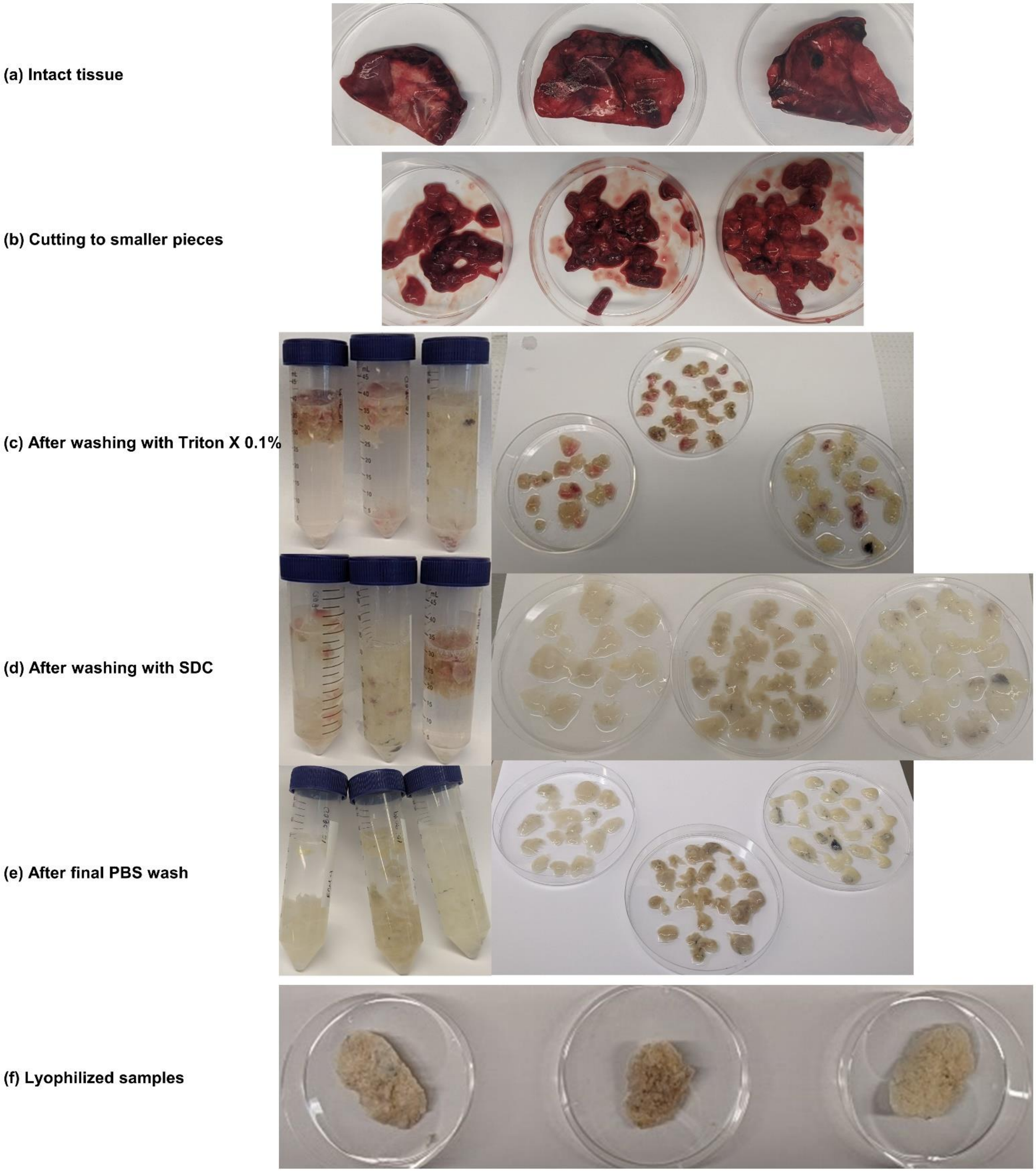

Figure S1: macroscopic images from various decellularization process steps

	Slope	$t_{1/2}$	$t_{95\%}$	t_{lag}
10.0 mg/mL vs. 12.5 mg/mL	ns	ns	ns	ns
10.0 mg/mL vs. 15.0 mg/mL	ns	ns	ns	ns
10.0 mg/mL vs. 17.5 mg/mL	ns	ns	ns	ns
10.0 mg/mL vs. 20.0 mg/mL	*	**	****	**
12.5 mg/mL vs. 15.0 mg/mL	ns	ns	ns	ns
12.5 mg/mL vs. 17.5 mg/mL	ns	ns	**	*
12.5 mg/mL vs. 20.0 mg/mL	*	*	****	**
15.0 mg/mL vs. 17.5 mg/mL	ns	ns	*	ns
15.0 mg/mL vs. 20.0 mg/mL	**	**	****	**
17.5 mg/mL vs. 20.0 mg/mL	ns	ns	**	ns

*Figure S2: A summary of statistical analysis and p-values for calculated turbidimetric gelation kinetics parameters in Table 1. * $p \leq 0.05$, ** $p \leq 0.01$, and **** $p \leq 0.0001$*

	G’	G’’
10.0 mg/mL vs. 12.5 mg/mL	ns	ns
10.0 mg/mL vs. 15.0 mg/mL	ns	ns
10.0 mg/mL vs. 17.5 mg/mL	*	*
10.0 mg/mL vs. 20.0 mg/mL	**	**
12.5 mg/mL vs. 15.0 mg/mL	ns	ns
12.5 mg/mL vs. 17.5 mg/mL	ns	ns
12.5 mg/mL vs. 20.0 mg/mL	*	*
15.0 mg/mL vs. 17.5 mg/mL	ns	ns
15.0 mg/mL vs. 20.0 mg/mL	ns	*
17.5 mg/mL vs. 20.0 mg/mL	ns	ns

*Figure S3: A summary of statistical analysis and p-values for G’ and G’’ in Figure 3e. * $p \leq 0.05$ and ** $p \leq 0.01$.*

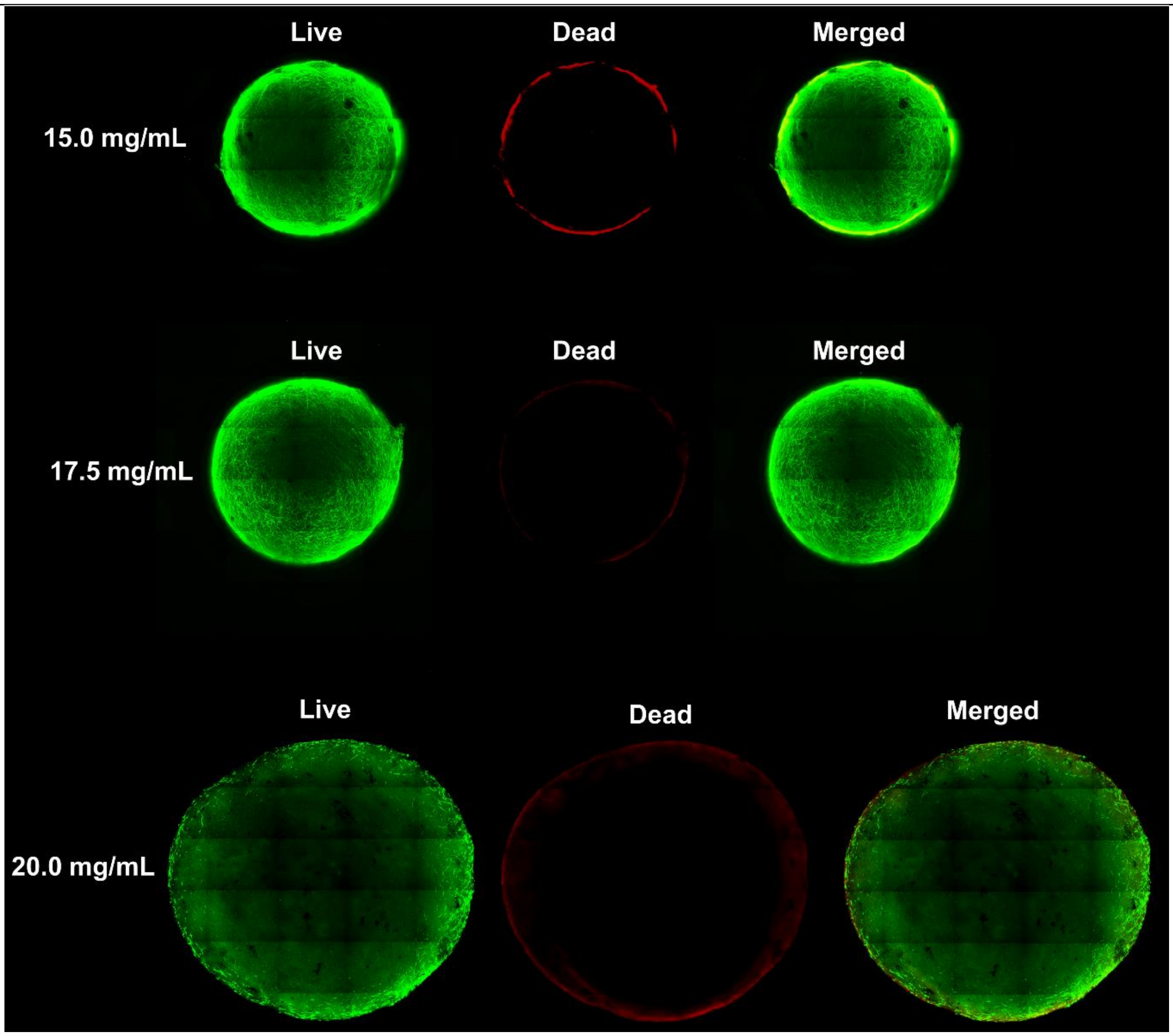

Figure S4: Viability assays of cells incubated inside dECM hydrogels using the live and dead assay.

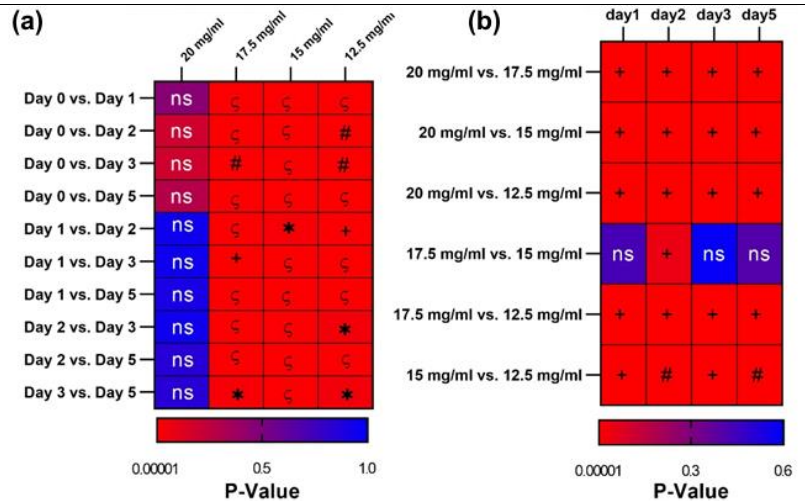

Figure S5: (a) heat map of the p-values for each hydrogel over five days: this graph shows if the change in the diameter of the hydrogel is significantly changed over time and (b) heat map of the p-values analysis for hydrogels compared to each other over five days of 3D culture. P-Values: ✱ denotes for p values smaller than 0.05, ς denotes for p values smaller than 0.005, # denotes for p values smaller than 0.001, + denotes for p values smaller than 0.0001.
